# Parental Social Comparisons and Parent–Child Closeness in Families with Preschool Children: The Roles of Mindful Parenting and Parents’ Education Anxiety

**DOI:** 10.3390/bs16071057

**Published:** 2026-06-25

**Authors:** Mingzhu Wang, Hui Liu, Xiang Yao, Xingrui Zhang, Yaming Lin, Mengjie Yu, Millicent Aziku

**Affiliations:** Faculty of Education, Shaanxi Normal University, Xi’an 710062, China; liuhui200105@163.com (H.L.); yaoxiang@snnu.edu.cn (X.Y.); jyxbzhangxingrui@snnu.edu.cn (X.Z.); edulinyaming@126.com (Y.L.); yumengjie0011@snnu.edu.cn (M.Y.); millyzi78@snnu.edu.cn (M.A.)

**Keywords:** parental social comparisons, parent-child closeness, mindful parenting, parents’ education anxiety

## Abstract

Chinese parents have a tendency to compare their children with peers. However, little is known about the implications of parental social comparisons for parent–child interactions. The current study investigated the associations between parental social comparisons and parent–child closeness among 317 Chinese parents of 5- to 6-year-old children. In addition, the mediating role of mindful parenting and the moderating role of parental education anxiety were examined. Results showed that parents’ upward contrast was negatively associated with parent–child closeness, upward identification was positively associated with parent–child closeness, whereas downward contrast and downward identification were unrelated to parent–child closeness. Additionally, mindful parenting mediated the relations of parental upward contrast and upward identification to parent–child closeness. Moreover, parents’ education anxiety moderated the negative association between upward contrast and mindful parenting, such that this association was stronger in parents with lower education anxiety levels. These findings highlight the importance of guiding parents to avoid non-constructive social comparison strategies and helping them manage their education anxiety, with potential implications for promoting positive parent–child interactions.

## 1. Introduction

People frequently compare themselves with others, a phenomenon known as social comparison. Social comparison refers to the psychological process through which individuals form self-evaluations of their abilities and opinions by comparing themselves to others in the absence of objective criteria (Festinger, 1954). In China, social comparison is relatively common because individuals in collectivistic cultures tend to possess an interdependent self-construal and rely on comparisons with others to define themselves (Baldwin & Mussweiler, 2018; Markus & Kitayama, 1991). The concept of social comparison has recently been introduced into the study of family education. Research has indicated that Chinese parents frequently engage in social comparisons by comparing their children’s performance with that of other children (e.g., Burman et al., 2025; Cao et al., 2025; B. Chen et al., 2021; H. Liu et al., 2025; Zhang & Yan, 2024). This tendency is known as parental social comparison.

Existing studies have primarily focused on parental social comparisons in adolescent families, but few have examined parents of young children. Unlike parents of adolescents who primarily compare their children’s academic performance with that of their peers, parents of young children make comparisons across a broader range of domains, including academic performance and nonacademic characteristics such as daily habits and manners (Zhang & Yan, 2024). Additionally, parent–child interactions during early childhood are highly frequent, as parents are continuously involved in children’s daily care and upbringing (Coe et al., 2024; Kakhki et al., 2022). As a result, parents have more opportunities to observe their children and compare them with their peers. The perceptions that parents form about their young children through social comparisons are more likely to manifest in their daily interactions, thereby affecting parent–child relationship (Jensen et al., 2018). Furthermore, preschool children have not yet developed mature cognitive and self-regulatory capacities (Goldenthal et al., 2025; Stuhr et al., 2020), making them particularly sensitive to parental evaluations and responses (McDermott et al., 2024). Interaction patterns established during this period not only influence the quality of the parent–child relationship, but also have implications for children’s interpersonal functioning, emotion regulation, and social adjustment (e.g., Fernandes et al., 2020; Kerr et al., 2021; X. Liu et al., 2025; Luo & Qiu, 2024). Therefore, it is important to investigate how social comparisons among parents of young children shape parent–child interactions. This study examined the associations between different types of parental social comparisons and parent–child closeness in families with preschool children. The underlying mechanisms were also explored.

### 1.1. Parental Social Comparisons and Parent–Child Closeness

According to social comparison theory (Masters & Keil, 1987), individuals evaluate their abilities and opinions by comparing themselves with others, which leads to changes in their attitudes and judgements. Specific to the family context, parents may adjust their evaluations of their children’s abilities and performance based on peer comparisons (Jensen & McHale, 2015), which may influence how they think and feel about their children. From a social cognition approach, social comparison can be viewed as a cognitive activity through which individuals process external social information (Bargh, 1996). In parent–child interactions, parents’ social comparisons are often triggered by exposure to information related to their children’s performance (Q. Yang et al., 2021). According to the social information processing model (Milner, 1993, 2003), when engaging in social comparisons, parents first attend to and become aware of information indicating how their children perform relative to their peers. Based on this comparison information, parents make internal and external attributions and form evaluations of their children’s current performance and future potential. These cognitive processes may directly influence parents’ perceptions of their children (Vogels & Perunovic, 2019), which may shape the climate of parent–child interactions, with implications for the emotional connection within the relationship (W. Li et al., 2024; Thai et al., 2019). Indeed, one study found that parents’ comparisons within the family (i.e., parents’ comparisons of siblings) were associated with differences in siblings’ perceptions of parent–child conflict (Jensen et al., 2018). Nevertheless, evidence concerning the association between parental comparisons of their children with peers outside the family and parent–child closeness is scarce.

Moreover, most existing studies conceptualize parental social comparison as a unidimensional construct and define it as a negative strategy involving blame and shame induction (e.g., Camras et al., 2012; Cao et al., 2025), but few have examined different forms of parental social comparisons. Based on the direction of social comparisons, previous research has distinguished between upward and downward comparisons, with the former referring to comparisons with superior others and the latter referring to comparisons with inferior others (Wheeler, 1966; Wills, 1981). Furthermore, when responding to comparison information, individuals may adopt contrast (i.e., focusing on differences with the comparison target) or identification (i.e., focusing on similarities with the comparison target) strategies (Blanton, 2001; Buunk & Gibbons, 2007; Collins, 1996). Accordingly, social comparisons can be classified into four types: upward contrast, upward identification, downward contrast, and downward identification. Most studies have categorized upward contrast and downward identification as negative strategies, whereas upward identification and downward contrast are generally regarded as positive strategies (e.g., Buunk & Brenninkmeijer, 2022; Esteves et al., 2021; Pérez-Torres, 2024; Van der Zee et al., 2000). However, recent studies have suggested that this dichotomy may vary across contextual and cultural settings. For example, in their study of social comparisons on social media, Yue et al. (2022) found that upward identification, which is typically considered an adaptive strategy, failed to show a significant association with perceived stress. Moreover, downward contrast was positively associated with stress, suggesting that it did not exhibit the expected protective effect on individuals’ emotional well-being. In addition, using a sample of Hispanic students, Huynh et al. (2026) found that downward identification, which is commonly regarded as a maladaptive strategy, was instead associated with higher self-confidence and lower levels of self-doubt. These inconsistent findings highlight the need for further research on social comparisons in diverse contexts and populations. Furthermore, research has suggested that parents also adopt these four strategies when comparing their children to others (Xing et al., 2023). When engaging in upward contrast, parents evaluate the discrepancy between their children and better-performing peers and tend to focus on areas in which their children fall short (H. Liu et al., 2025). In contrast, parents who adopt upward identification view better-performing peers as role models and maintain positive expectations regarding their children’s future improvement (Rheu et al., 2023). When engaging in downward contrast, parents attend to the shortcomings of lower-performing peers and derive psychological comfort from their own children’s relative strengths (Q. Yang et al., 2021). By contrast, parents who adopt downward identification tend to recognize similarities between their children and lower-performing peers, viewing the challenges faced by other children as potential risks that their own children may also encounter (Q. Wang et al., 2023). However, the associations between different types of parental social comparisons and parent–child closeness remain to be examined.

### 1.2. Parental Social Comparisons, Mindful Parenting, and Parent–Child Closeness

To gain a deeper understanding of the relationship between parental social comparisons and parent–child closeness, it is necessary to explore the underlying mechanisms. The social information processing model (Milner, 1993, 2003) proposes that the cognitive processing of information consists of three stages, in which individuals perceive the information, interpret and evaluate its meaning, and integrate the information to guide response selection. These cognitive activities subsequently influence the behavioral stage of response implementation. Based on this perspective, parents’ comparisons of their children with peers may serve as an important basis for parenting decisions. By engaging in social comparisons, parents become aware of differences between their children and their peers, and such comparison-based information provides important cues for parents to evaluate their children. Parents then integrate social comparison information with other situational information and child-specific characteristics when making parenting decisions. These decisions, in turn, are translated into specific parenting practices and behaviors. Existing studies have demonstrated that parents’ processing of child-related information is linked with their implementation of parenting behaviors (e.g., Dănilă et al., 2023; Sawrikar et al., 2020), which in turn are associated with the relationship between the parent and the child (Lanjekar et al., 2022). Therefore, parenting behaviors may mediate the relation between parental social comparisons and parent–child closeness. This study proposed that parental social comparisons may be associated with mindful parenting, which is characterized by full attentiveness to and non-judgmental acceptance of the child (Duncan, 2007), and may in turn be related to parent–child closeness.

Different social comparison strategies may be differentially associated with mindful parenting. Parents who engage in upward contrast or downward identification tend to be more attentive to children’s weaknesses and develop negative evaluations of their children (Xing et al., 2023). When making parenting choices, these parents may integrate information regarding social competition while ignoring their children’s psychological needs. As a result, they might struggle to attend to the present moment in parent–child interactions and display lower responsiveness (Azar et al., 2008). That is, parental upward contrast and downward identification may be associated with lower levels of mindful parenting. Conversely, parents who employ upward identification and downward contrast are better able to recognize their children’s strengths and formulate positive evaluations of their abilities (Xing et al., 2023). These parents may effectively integrate information about their children’s developmental potential, and become more motivated to listen attentively, accept, and respond sensitively to their children. Thus, parental upward identification and downward contrast may be linked to greater mindfulness in parenting. Furthermore, a number of studies have demonstrated that mindful parenting contributes to the quality of parent–child communication and strengthens the intimate connection between the parent and the child (e.g., Chan et al., 2022; Y. Chen et al., 2019; W. Yang et al., 2022). Taken together, mindful parenting may play a mediating role in the relationship between parental social comparisons and parent–child closeness.

### 1.3. The Role of Parents’ Education Anxiety

The social information processing model (Milner, 2003) emphasizes the importance of considering the moderating role of parental affective states and stress in cognitive processing activities. When parents experience negative affect and when stress increases, they may attend selectively to children’s behavior, and develop increasingly distorted perceptions, interpretations, and expectations regarding their children. Moreover, high levels of negative affect and stress may reduce parents’ ability to adequately integrate child-related information when selecting an appropriate response. Consequently, biases in these cognitive processes may ultimately influence the implementation of parenting behaviors. From this perspective, the association between parental social comparisons and parenting behaviors may vary as a function of parents’ emotions. Indeed, there has been some empirical evidence supporting the interaction between parents’ cognitive activities and their negative affect on parenting behaviors (e.g., González & Trujillo, 2024; Milner et al., 2019). However, research on this topic is still limited. Among Chinese parents, education anxiety is a commonly experienced emotion (G. Chen et al., 2022). It refers to the tension and worry arising from uncertainty within the education context and the unpredictability of children’s future development (Niu et al., 2025; M. Wang et al., 2025). Parents with higher education anxiety levels tend to hold pessimistic predictions about children’s future development (Q. Liu et al., 2022). They may worry that their children will fall behind their peers or miss important developmental opportunities (Hong et al., 2025). At the same time, anxious parents also hold unrealistically high expectations for their children and find it difficult to accept their failures (Wu et al., 2022). When experiencing high levels of education anxiety, parents may become less emotionally engaged with their children and instead rely more heavily on excessive behavioral control (K. Liu et al., 2026). In contrast, parents with lower education anxiety levels tend to view their children’s weaknesses more rationally and avoid overemphasizing educational outcomes (M. Wang et al., 2025). Because education anxiety may influence how parents perceive, interpret, and form expectations about their children, this study proposes that it may play a moderating role in the associations between parental social comparisons and mindful parenting.

Specifically, when focusing on children’s differences from better-performing peers (i.e., upward contrast) or similarities with lower-performing peers (i.e., downward identification), parents with lower education anxiety may be less likely to interpret such social comparison information as threatening (Gallagher & Cartwright-Hatton, 2009; Zhou & Li, 2022). In such cases, they are less inclined to adopt performance-oriented goals in their parenting (Haimovitz & Dweck, 2016), a tendency that aligns with maintaining a more non-judgmental attitude toward their children. That is, lower levels of education anxiety may weaken the associations of parents’ upward contrast and downward identification with mindful parenting. In contrast, when focusing on children’s similarities with better-performing peers (i.e., upward identification) or differences from lower-performing peers (i.e., downward contrast), parents with lower education anxiety may evaluate this information more positively (Laifer et al., 2023). They tend to adopt mastery-oriented goals that emphasize supporting their children’s improvement (Haimovitz & Dweck, 2016) and show greater motivation to listen to and accept their children during parent–child interactions (Wu et al., 2022). Hence, lower levels of education anxiety may enhance the positive associations of parents’ upward identification and downward contrast with mindful parenting. To sum up, parental education anxiety may moderate the relations between different types of parental social comparisons and mindful parenting.

### 1.4. The Present Study

Guided by Milner’s (2003) social information processing model, the present study aimed to investigate the direct and indirect effects of parents’ social comparisons on parent–child closeness in early childhood and the moderating role of parental education anxiety. We proposed three hypotheses. First, parental upward contrast and downward identification would be negatively associated with parent–child closeness, whereas upward identification and downward contrast would be positively associated with parent–child closeness (H1). Second, mindful parenting was expected to mediate the relations between parental social comparisons and parent–child closeness (H2). Third, parental education anxiety was hypothesized to moderate the relations between social comparisons and mindful parenting (H3).

## 2. Materials and Methods

### 2.1. Participants and Procedure

Participants were 317 parents (Mage = 37.22 years, SD = 5.17), including 246 mothers and 71 fathers, who took part in an early childhood parenting project. All participants had at least one child aged 5–6 years, 53.0% of whom were girls. They were recruited from four kindergartens located in urban areas of China, including two kindergartens in central China (i.e., Henan and Shanxi provinces) and two in western China (i.e., Guizhou and Yunnan provinces). Most parents had received higher education, with 15.1% holding a junior college diploma, 56.8% a bachelor’s degree, and 16.4% a graduate degree, whereas 11.7% completed only secondary education. The data were collected through an online survey platform. Kindergarten teachers helped distribute the survey link to families in their class and invited one primary caregiver (mother or father) from each family to participate. Participation was voluntary, and parents who provided informed consent completed the survey and were subsequently compensated.

### 2.2. Measures

#### 2.2.1. Parental Social Comparisons

Parental social comparisons were assessed with a scale used by Xing et al. (2023), which was originally developed by Van der Zee et al. (2000). In Xing et al.’s (2023) study, adolescents reported their perceptions of parents’ social comparisons regarding their academic achievement. This study modified the stem of each item to read “When other children perform better/worse than my child” to make the scale suitable for parents’ self-reports. Furthermore, the academic-related phrases in the original scale (e.g., “school record”) were replaced with “performance” to assess parents’ social comparisons of their children’s overall performance. The revised scale was reviewed by a panel consisting of two experts in early childhood education and two graduate students. The reviewers were asked to evaluate whether the wording of the items was appropriate and whether the content was suitable for parents of young children. Based on their feedback and suggestions, several items were reworded, resulting in the final version of the scale used in this study. The scale consists of twelve items, with three items in each of four dimensions: upward contrast (e.g., “it is threatening to notice that my child is not doing well”), upward identification (e.g., “I am happy and realize that my child can improve”), downward contrast (e.g., “I realize how well my child is doing”), and downward identification (e.g., “I fear that my child’s performance will become worse like that in the future”). Each item was rated on a 5-point scale ranging from 1 (not at all true) to 5 (very true). Prior to the main data collection, a pilot study involving 148 participants (115 mothers and 33 fathers) was conducted to examine the psychometric properties of the revised scale. Confirmatory factor analysis indicated that this scale had good construct validity (*χ*^2^/*df* = 1.98, CFI = 0.95, TLI = 0.93, RMSEA = 0.08, SRMR = 0.07). The criterion-related questionnaire used in this study was adapted from the instrument developed by Haimovitz and Dweck (2016). This questionnaire consisted of 5 items assessing parents’ performance-oriented responses to children’s failure (α = 0.70) and 5 items assessing parents’ learning-oriented responses to children’s failure (α = 0.75). Correlation analyses showed that parental upward contrast, downward identification, and downward contrast were positively correlated with performance-oriented responses, whereas upward identification and downward contrast were positively correlated with learning-oriented responses. These findings indicated that the revised scale used in this study demonstrated good criterion-related validity. In the main study, the Cronbach’s *α*s for the four dimensions were 0.89, 0.90, 0.91, and 0.92, respectively.

#### 2.2.2. Parent–Child Closeness

The closeness subscale of the Child–Parent Relationship Scale—Short Form (Driscoll & Pianta, 2011) was used to assess parent–child closeness. The Chinese version of this scale was translated by Deng (2013). This scale comprises seven items (e.g., “My child values his/her relationship with me”) rated on a 5-point scale ranging from 1 (definitely does not apply) to 5 (definitely applies). The mean of the seven items was taken, with higher numbers indicating a closer parent–child relationship. The Cronbach’s *α* was 0.92 in this study.

#### 2.2.3. Mindful Parenting

The Inter-personal Mindfulness in Parenting scale, developed by Duncan (2007) and revised by Y. Chen et al. (2019), was used to measure mindful parenting. It consists of nine items concerning parents’ awareness and present-centered attention (e.g., “I notice how changes in my child’s mood affect my mood”), non-judgmental acceptance (e.g., “I listen carefully to my child’s ideas, even when I disagree with them”), and non-reactivity (e.g., “When I’m upset with my child, I notice how I am feeling before I take action”). Items were rated on a 5-point scale ranging from 1 (not at all true) to 5 (very true). A global score was obtained by averaging the nine items, with higher scores indicating greater mindfulness in parenting. In this study, the Cronbach’s *α* was 0.77.

#### 2.2.4. Parental Education Anxiety

Parental education anxiety was assessed using eleven items drawn from the instrument developed by M. Wang et al. (2025), which describe parents’ worries about their children’s school readiness and learning virtues (e.g., “I worry that my child can’t develop good learning habits”). Participants rated each item on a 5-point scale ranging from 1 (never) to 5 (always). The mean score of the eleven items was calculated, with higher scores indicating higher levels of education anxiety. The Cronbach’s *α* in this study was 0.96.

### 2.3. Data Analyses

Descriptive statistics and correlation analyses were conducted with SPSS 27.0. A mediation model was estimated using Mplus 7.4 to examine the mediating role of mindful parenting in the associations between parental social comparisons and parent–child closeness. Covariates, including child age, parent gender, parent education level, and number of children, were controlled for in the model. Child gender was not included as a covariate because it was unrelated to all the main study variables. The bootstrap method with 5000 resamples was used to evaluate the significance of the indirect effects. If the 95% confidence intervals (CIs) did not include 0, the indirect effects were considered significant at *p* < 0.05. To test whether education anxiety moderated the associations between parental social comparisons and mindful parenting, parental education anxiety and the interaction terms between education anxiety and each type of parental social comparisons were included as predictors of mindful parenting in the mediation model. Each variable was mean-centered prior to creating the interaction terms. The analyses were performed using the robust maximum likelihood estimation.

## 3. Results

### 3.1. Common Method Bias Test

To reduce the potential influence of common method bias, this study employed several procedural controls, including anonymous questionnaire administration and the use of reverse-coded items for part of the questionnaire. In addition, Harman’s single-factor test was conducted to statistically assess common method bias. The results showed that seven factors had eigenvalues greater than 1, and the first factor accounted for 28.19% of the total variance, which was below the critical threshold of 40%. These findings suggest that common method bias was not a serious concern in this study.

### 3.2. Descriptive Statistics and Correlation Analysis

Descriptive statistics and bivariate correlations for the main study variables are presented in Table 1. Parents’ upward contrast and downward identification were negatively correlated with mindful parenting and parent–child closeness, whereas upward identification and downward contrast were positively correlated with mindful parenting and parent–child closeness. Mindful parenting had a positive association with parent–child closeness. Parental education anxiety was negatively linked with parent–child closeness.

### 3.3. Direct and Indirect Effects

To investigate how parental social comparisons were directly and indirectly associated with parent–child closeness, a simple regression model and a mediation model were conducted with Mplus. Throughout this paper, the term “effects” was used to denote statistical effects rather than causal effects. First, a simple regression model was estimated to examine the total effects of parental social comparisons on parent–child closeness, controlling for covariates. The model was saturated and showed a perfect fit to the data. Results showed that parental upward contrast was negatively related to parent–child closeness (*β* = −0.15, *p* < 0.05), and upward identification was positively correlated with parent–child closeness (*β* = 0.29, *p* < 0.001). However, downward contrast (*β* = 0.05, *p* > 0.05) and downward identification (*β* = −0.07, *p* > 0.05) were unrelated to parent–child closeness. Therefore, H1 was partially supported.

**Figure 1:**
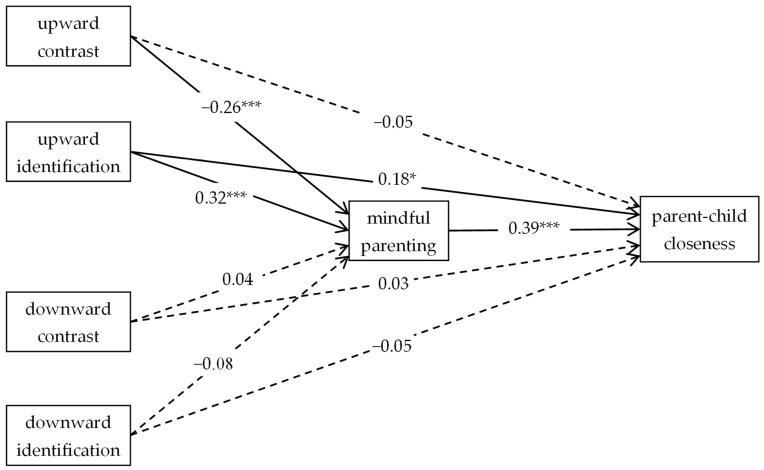
A mediation model depicting the indirect effects of parental social comparisons on parent–child closeness through mindful parenting. Note. Solid lines indicate significant paths, and dotted lines indicate nonsignificant paths. Standardized coefficients are reported. * *p* < 0.05, *** *p* < 0.001. The same in Figure 2.

Next, mindful parenting was included as a mediator in the model to examine whether it statistically accounted for the associations between parental social comparisons and parent–child closeness (see Figure 1). 

The model showed a good fit to the data, *χ*^2^/*df* = 1.82, CFI = 0.99, TLI = 0.93, RMSEA = 0.05, SRMR = 0.02. Results indicated that parental upward contrast was negatively associated with mindful parenting (*β* = −0.26, *p* < 0.001), upward identification was positively associated with mindful parenting (*β* = 0.32, *p* < 0.001), whereas downward contrast (*β* = 0.04, *p* > 0.05) and downward identification (*β* = −0.08, *p* > 0.05) were unrelated to mindful parenting. Moreover, mindful parenting was linked with higher levels of parent–child closeness (*β* = 0.39, *p* < 0.001). Bootstrapping indicated that both upward contrast (indirect effect = −0.10, 95% CI [−0.16, −0.04]) and upward identification (indirect effect = 0.12, 95% CI [0.07, 0.18]) were indirectly associated with parent–child closeness through mindful parenting. However, the indirect associations of downward contrast (indirect effect = 0.01, 95% CI [−0.03, 0.06]) and downward identification (indirect effect = −0.03, 95% CI [−0.08, 0.02]) with parent–child closeness through mindful parenting were not significant. Hence, H2 was also partially supported.

### 3.4. Moderating Effect of Parental Education Anxiety

**Figure 2:**
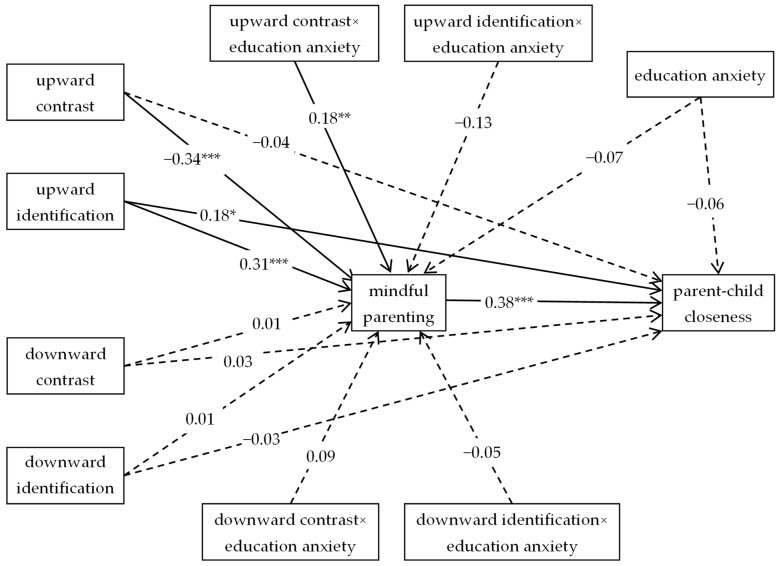
A moderated mediation model depicting the moderating role of parental education anxiety in the associations between parental social comparisons and parent–child closeness via mindful parenting. * *p* < 0.05, ** *p* < 0.01, *** *p* < 0.001.

To examine the potential moderating effect of parental education anxiety in the paths from parental social comparisons to mindful parenting, a moderated mediation model was examined (see Figure 2).

The model showed a good fit to the data, *χ*^2^/*df* = 1.11, CFI = 0.99, TLI = 0.99, RMSEA = 0.02, SRMR = 0.01. The interaction of parental upward contrast and education anxiety on mindful parenting was significant (*β* = 0.18, *p* < 0.01), which was consistent with H3. However, the interaction effects between upward identification and education anxiety (*β* = −0.13, *p* > 0.05), downward contrast and education anxiety (*β* = 0.09, *p* > 0.05), and downward identification and education anxiety (*β* = −0.05, *p* > 0.05) on mindful parenting were non-significant. Therefore, education anxiety only moderated the relation between upward contrast and mindful parenting.

**Figure 3:**
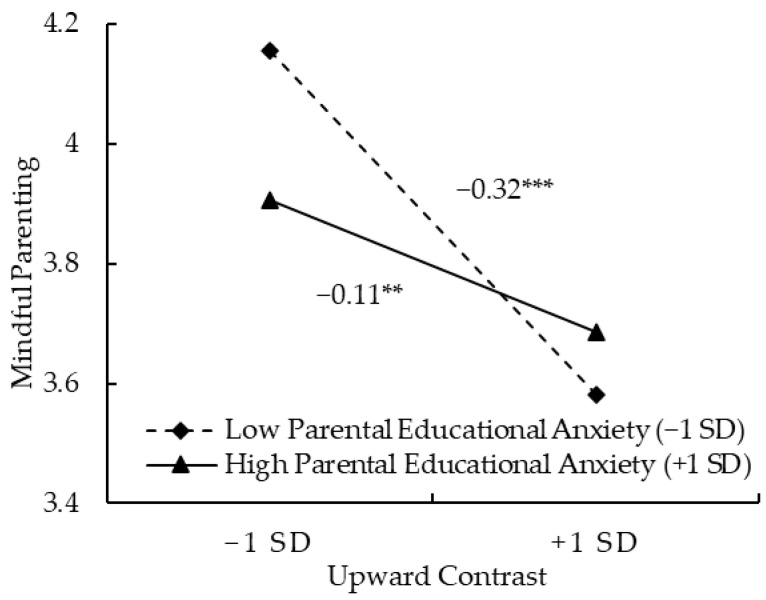
Mindful parenting as a function of upward contrast. Low upward contrast is 1 SD below mean upward contrast; high upward contrast is 1 SD above mean upward contrast. Low education anxiety is 1 SD below mean education anxiety; high education anxiety is 1 SD above mean education anxiety. ** *p* < 0.01, *** *p* < 0.001.

A simple slope test was conducted to further reveal the moderating effect of parental education anxiety. As shown in Figure 3, the negative association between parental upward contrast and mindful parenting was weaker among parents with higher levels of education anxiety (simple slope = −0.11, *t* = −2.57, *p* < 0.01), whereas this association was stronger among parents with lower levels of education anxiety (simple slope = −0.32, *t* = −4.08, *p* < 0.001).

## 4. Discussion

Due to intense social competition, Chinese parents often compare their children with peers to evaluate their performance and their likelihood of success in competitive contexts. Although parental social comparisons have received scholarly attention, previous studies have primarily focused on families with adolescents (e.g., Cao et al., 2025; B. Chen et al., 2021), with limited research on families with preschool children. Additionally, most existing studies have investigated the implications of parental social comparisons for child outcomes, but few have examined their associations with parent–child interactions. Furthermore, existing research has predominantly examined the negative aspects of parental social comparisons, with comparatively little attention devoted to their positive aspects. The current study addressed these research gaps by examining how different types of parental social comparisons were associated with parent–child closeness in early childhood. Results showed that the two types of upward comparisons were significantly associated with parent–child closeness, whereas downward comparisons and parent–child closeness were not significantly related. Mindful parenting served as a mediator in the links between parental upward comparisons and parent–child closeness. Additionally, the negative relation between upward contrast and mindful parenting was stronger among parents with lower education anxiety levels.

### 4.1. The Associations Between Parental Social Comparisons and Parent–Child Closeness

Consistent with the hypothesis, this study found that parental upward contrast was negatively correlated with parent–child closeness, whereas parental upward identification was positively correlated with parent–child closeness. These results suggested that parents’ social comparisons may be associated with their evaluations and attitudes toward their children, as reflected in the emotional closeness in the parent–child relationship. Previous research has established a link between parents’ social comparisons of sibling and parent–child relationship (Jensen et al., 2018). This study contributes to the literature by extending these findings to parents’ comparisons of their children with their peers outside the family. Because of China’s intense educational competition, it is more common for Chinese parents to make peer comparisons (Zhang & Yan, 2024). Thus, investigating parents’ peer comparisons may be particularly important for understanding the quality of parent–child relationship. Furthermore, this study extended prior work by examining various types of parental social comparisons. The results indicate that different types of parental social comparisons are differentially associated with parent–child closeness. Parents who make upward contrast tend to place greater emphasis on children’s external performance than on their emotional needs. In the Chinese cultural context, many parents endorse the belief that children should not “fall behind at the starting line” (G. Chen et al., 2022; Tian & Jing, 2021). When parents perceive their children as lagging behind better-performing peers, they may interpret the child’s performance in a negative manner and display more criticism and unrealistic expectations during parent–child interactions (Zhang & Yan, 2024). Such interaction patterns are often associated with lower levels of parent–child closeness (X. Wang & Wang, 2026). In contrast, parents who engage in upward identification believe that their children have the potential to overcome their shortcomings and make progress, which aligns with the Chinese cultural emphasis on improvement through continual effort (Pomerantz et al., 2020). When parents hold positive views of their children, they are more motivated to be warm and caring (Xing et al., 2023) and to maintain close parent–child relationships. These findings address limitations of previous studies that regarded parental social comparisons as a unidimensional construct largely characterized by negative aspects.

However, the two types of parental downward comparisons were unrelated to parent–child closeness after controlling for upward comparisons, which was inconsistent with the hypothesis. First, parents’ downward contrast showed no association with parent–child closeness. Although downward contrast is considered a strategy that offers psychological comfort (Wills, 1981), our findings suggest that such comfort might be weak. Parents may not necessarily show stronger affective attitudes toward their children simply because they recognize that their children are not currently lagging behind. Given the cultural importance of self-improvement, Chinese parents tend to emphasize children’s failure and downplay their success (Pomerantz et al., 2020). Even when parents recognize that their children are performing better than lower-performing peers, such evaluations may not necessarily translate into highly positive parental attitudes. Parents may still view their children’s performance as inadequate because it does not meet their standards for excellence. Therefore, parents who engage in downward contrast may not necessarily develop closer emotional bonds with their children. Second, no significant association was found between downward identification and parent–child closeness. Downward identification reflects one’s pessimistic tendency towards social comparison information (Van der Zee et al., 2000). When parents identify their children with peers who are performing worse, they may fear that their children are also likely to perform poorly in the future (Q. Wang et al., 2023). Consequently, parents may perceive other children’s poor performance as a warning sign (Ng et al., 2017) and become aware of potential problems in their own children. Nevertheless, downward identification does not imply that parents devalue their children’s actual performance or develop negative attitudes toward them, and is thus unrelated to the closeness of parent–child relationship.

### 4.2. The Mediating Role of Mindful Parenting

As expected, mindful parenting mediated the relations of parental upward contrast and upward identification to parent–child closeness. These findings provide empirical support for the social information processing model (Milner, 1993). Parents’ processing of social comparison information related to their children is associated with their behavioral choices and practices in parenting, which in turn are linked with parent–child relationship. Specifically, parents who engage in upward contrast may evaluate their children’s performance more negatively (Xing et al., 2023). When parents attend more to children’s shortcomings than to their current psychological needs, lower levels of sensitive support also tend to be observed in their interactions with children. Additionally, upward contrast may reinforce parents’ conditional regard (Haines & Schutte, 2023). That is, parental approval is contingent upon children fulfilling parents’ expectations (Ng & Wei, 2020), which conflicts with the nonjudgmental acceptance characteristic of mindful parenting. Moreover, East Asian cultures place great value on self-criticism, and individuals in these societies are more likely to focus on their weaknesses (Pomerantz et al., 2020). Shaped by these cultural beliefs, Chinese parents may pay closer attention to whether their children meet social expectations or fall behind their peers. When engaging in upward contrast, Chinese parents may develop negative perceptions of their children and find it difficult to remain attentive to and accepting of them. Therefore, parents who engage in upward contrast tend to exhibit lower levels of mindful parenting, making it difficult for them to develop close relationships with their children (Y. Chen et al., 2019).

In contrast, parents who engage in upward identification focus on their children’s similarities with better-performing peers and believe their children have the potential to achieve progress (Xing et al., 2023). These parents may be less inclined to criticize their children and may display greater acceptance and affection (Ren et al., 2025), which are consistent with the principles of mindful parenting. In addition, parents’ positive beliefs about their children may be linked to more positive self-evaluations (Fierloos et al., 2023). This may be reflected in parents’ greater engagement and patience in parent–child interactions (Buchanan et al., 2022), allowing them to listen to their children with full attention and respond sensitively to children’s needs. From a cultural perspective, upward identification reflects Chinese parents’ tendency to view other children who are performing better as role models for encouraging their own children to improve. Influenced by the cultural belief that abilities can be developed through effort, parents often perceive other children’s positive developmental outcomes as attainable goals (Wei et al., 2020). Consequently, when parents adopt upward identification, they are more likely to hold optimistic expectations for their children, which are typically associated with greater dedication to parenting (Burnette et al., 2025). Therefore, upward identification is associated with higher levels of mindful parenting, which in turn is related to parent–child closeness.

Nevertheless, mindful parenting was not found to mediate the relations of parental downward contrast and downward identification to parent–child closeness, given that downward comparisons were unrelated to mindful parenting after adjusting for upward comparisons. According to the social comparison perspective, upward comparisons are often driven by self-improvement motives (Buunk & Gibbons, 2007; Wood, 1989). In the parenting context, parents who make upward comparisons usually expect their children to make progress, and are therefore more motivated to take action for their children (Rheu et al., 2023). Conversely, downward comparisons are usually passive (Buunk & Gibbons, 2007), rather than involving parents actively seeking peers who perform worse than their children. Parents may be less inclined to change their parenting behaviors because of downward comparison results. Hence, after accounting for upward comparisons, the associations between parents’ downward comparisons and mindful parenting turned nonsignificant.

### 4.3. The Moderating Role of Parental Education Anxiety

This study showed that parental education anxiety moderated the relation between upward contrast and mindful parenting. These results suggest that parents’ processing of child-related information interacts with their anxiety about children’s education in shaping parenting behaviors. Specifically, the negative association between parents’ upward contrast and mindful parenting became stronger as their education anxiety levels decreased, which was inconsistent with the buffering pattern proposed in the hypothesis. According to the hypothesis, low levels of education anxiety serve as a protective factor in parent–child interactions and may help mitigate the negative effects of high levels of upward contrast on mindful parenting. That is, even when parents have high levels of upward contrast, they may still exhibit relatively high levels of mindful parenting if they experience low levels of education anxiety. However, our findings indicated that parents showed a high level of mindful parenting under conditions where upward contrast and education anxiety were both low. One possible explanation is that both upward contrast and education anxiety may deplete parents’ psychological resources. Upward contrast may involve parents paying greater attention to their children’s shortcomings (Cao et al., 2025), potentially placing greater cognitive demands on them. Education anxiety reflects parents’ stressful feelings regarding children’s education (M. Wang et al., 2025) and may occupy their emotion regulation resources. Parents may be more likely to possess sufficient psychological resources to be fully attentive, responsive, and accepting in parent–child interactions when both upward contrast and education anxiety are at low levels, thus supporting optimal parental functioning (Belsky, 1984). In addition, the weaker negative association between parental upward contrast and mindful parenting among parents with higher education anxiety levels may be understood within the sociocultural context of China. Chinese culture places emphasis on parents’ responsibility for their children’s education, and children’s development is often regarded as a central family obligation (Ng & Wei, 2020). Therefore, parental education anxiety may involve not only emotional distress, but also a heightened sense of parental responsibility and continued educational investment (Niu et al., 2025; Xin & Yu, 2024). When engaging in upward contrast and perceiving their children as lagging behind better-performing peers, highly anxious parents may still maintain their involvement in their children’s development. Such involvement may reflect parental attention and interaction with their children, thereby buffering the negative association between upward contrast and mindful parenting. Nevertheless, this finding contradicted the hypothesized buffering pattern and was not fully aligned with Milner’s (2003) theoretical perspective. The explanations proposed above should be treated as speculative. Future studies using more diverse samples are needed to examine the generalizability of this finding.

Inconsistent with the assumption, the relation between parental upward identification and mindful parenting did not vary as a function of education anxiety. Parents who engage in upward identification generally hold a positive view of their children (Xing et al., 2023), which encourages them to be actively involved in their children’s growth. Even when they feel anxious about their children’s education, they are less likely to interpret this anxiety as pressure or threat. Instead, they may be motivated to engage constructively in the parenting process (Xin & Yu, 2024). Therefore, parental education anxiety did not moderate the association between upward identification and mindful parenting. Moreover, the moderating roles of parental education anxiety on the associations between the two types of downward comparisons (i.e., downward contrast and downward identification) and mindful parenting were not significant. As noted above, there were no significant associations between downward comparisons and mindful parenting. These findings suggest that parents’ positive perceptions of their children arising from downward comparisons do not necessarily motivate them to adopt more constructive parenting behaviors. Education anxiety is typically associated with parents’ sensitivity to information regarding their children’s performance (Shek et al., 2025), and it may be linked to how parents regulate their parenting behaviors (M. Wang et al., 2025). However, because downward comparisons do not appear to establish a stable motivational pathway toward mindful parenting, education anxiety may have limited capacity to strengthen or weaken this relationship.

### 4.4. Limitations and Future Research

Several limitations of this study should be noted. First, the cross-sectional design used in this study does not allow for causal inferences. Although the present study proposed associations among parental social comparisons, mindful parenting, and parent–child closeness based on relevant theories, these associations may be bidirectional. For instance, mindful parenting may also influence parents’ tendency to engage in social comparisons. Research has shown that parents who adopt mindful parenting tend to report higher levels of parenting self-efficacy (Fereydooni et al., 2020). When parents believe they are capable of handling the challenges of child-rearing, they become less dependent on external evaluations (Burman et al., 2025). That is, they are less likely to rely on social comparisons to evaluate their parenting competence or assess their children’s development. Therefore, future research could employ longitudinal designs to examine the potential bidirectional relations among the study variables. Second, participants in this study were recruited from urban areas, and most of the parents had relatively high levels of education. As a result, the findings may be more applicable to urban middle-class families than to other populations. Rural communities tend to be characterized by closer social connections, whereas urban families are exposed to greater social competition (H. Yang, 2014). Therefore, rural and urban parents may differ in the ways they engage in social comparisons and the extent to which they make such comparisons. In addition, parents from different regions and socioeconomic backgrounds usually hold different parenting beliefs and adopt different parent–child interaction patterns (Han et al., 2023; X. Li et al., 2020). Hence, the associations among parental social comparisons, mindful parenting, and parent–child closeness may vary across different family contexts. Future studies could include families from both urban and rural areas, as well as from a wider range of socioeconomic backgrounds. Comparing families from different regions and diverse backgrounds would contribute to a deeper understanding of the research questions examined in the present study. Third, the present study primarily involved mothers, while fathers were underrepresented in the sample, which may limit the generalizability of the findings. Previous research has suggested that mothers and fathers often differ in their parenting roles (Aram et al., 2026; Y. Liu et al., 2022). Mothers typically assume greater responsibility for daily caregiving and are more concerned with whether their children are meeting age-appropriate developmental expectations. Fathers are more likely to take on roles related to children’s socialization and to place greater emphasis on children’s development of rule awareness. Hence, when engaging in social comparisons, mothers and fathers may focus on different developmental domains, and such comparisons may arise in different contexts. Future research could collect data from both parents to better understand how mothers’ and fathers’ social comparisons relate to parent–child interactions. Fourth, data in this study were collected exclusively from parent reports, which may be susceptible to common method bias and social desirability. Although statistical tests indicated that common method bias was not a serious concern in this study, its potential influence could not be completely ruled out. Previous research has shown that relying on self-reports from a single respondent can lead to overestimated associations among variables (Yao & Xu, 2024). Additionally, parents’ self-reports of their parenting behaviors do not always match observed parent–child interactions (Potharst et al., 2021). When reporting on parenting behaviors and parent–child relationships, parents may respond in socially desirable ways, which could bias the findings. Future studies could include child reports or observation methods to assess parent–child interaction variables, thereby enhancing the validity of the findings.

## 5. Conclusions

In conclusion, the present study explored the associations between four different types of parental social comparisons and parent–child closeness, as well as the underlying mechanisms. These findings contribute to a more comprehensive understanding of parental social comparisons. Moreover, this study extends social comparison theory into the field of family education, offering a new perspective for understanding parenting behaviors and parent–child relationship. Results suggest that the use of upward identification, relative to upward contrast, is associated with more positive parent–child interactions. These findings provide insights for parents to use more constructive strategies and recognize their children’s potential for improvement when comparing children with better-performing peers. It may also be beneficial to help parents set more realistic educational expectations and better manage education anxiety.

## Figures and Tables

**Table 1:** Means, standard deviations, and correlations among study variables.

	*M*	*SD*	1	2	3	4	5	6
1. Upward contrast	1.85	0.89						
2. Upward identification	4.30	0.75	−0.34 ***					
3. Downward contrast	3.89	0.95	−0.05	0.42 ***				
4. Downward identification	2.11	1.03	0.61 ***	−0.18 **	0.06			
5. Mindful parenting	3.87	0.58	−0.42 ***	0.44 ***	0.19 ***	−0.28 ***		
6. Parental education anxiety	2.66	1.01	0.46 ***	−0.21 ***	−0.09	0.54 ***	−0.31 ***	
7. Parent–child closeness	4.54	0.58	−0.31 ***	0.40 ***	0.20 ***	−0.24 ***	0.53 ***	−0.27 ***

Note. ** *p* < 0.01, *** *p* < 0.001.

## Data Availability

Anonymized data, analysis syntax, and study materials will be made available from the corresponding author upon reasonable request.
